# Decentralising HIV treatment in lower- and middle-income countries

**DOI:** 10.1590/1516-3180.20141326T2

**Published:** 2014-09-02

**Authors:** Tamara Kredo, Nathan Ford, Folasade B. Adeniyi, Paul Garner

## Abstract

**BACKGROUND:**

Policy makers, health staff and communities recognise that health services in lower- and middle-income countries need to improve people's access to HIV treatment and retention to treatment programmes. One strategy is to move antiretroviral delivery from hospitals to more peripheral health facilities or even beyond health facilities. This could increase the number of people with access to care, improve health outcomes, and enhance retention in treatment programmes. On the other hand, providing care at less sophisticated levels in the health service or at community-level may decrease quality of care and result in worse health outcomes. To address these uncertainties, we summarised the research studies examining the risks and benefits of decentralising antiretroviral therapy service delivery.

**OBJECTIVES:**

To assess the effects of various models that decentralised HIV treatment and care to more basic levels in the health system for initiating and maintaining antiretroviral therapy.

**METHODS:**

*Search methods:* We conducted a comprehensive search to identify all relevant studies regardless of language or publication status (published, unpublished, in press, and in progress) from 1 January 1996 to 31 March 2013, and contacted relevant organisations and researchers. The search terms included "decentralisation", "down referral", "delivery of health care", and "health services accessibility". *Selection criteria:* Our inclusion criteria were controlled trials (randomised and non-randomised), controlled-before and after studies, and cohorts (prospective and retrospective) in which HIV-infected people were either initiated on antiretroviral therapy or maintained on therapy in a decentralised setting in lower- and middle-income countries. We define decentralisation as providing treatment at a more basic level in the health system to the comparator. *Data collection and analysis:* Two authors applied the inclusion criteria and extracted data independently. We designed a framework to describe different decentralisation strategies, and then grouped studies against these strategies. Data were pooled using random-effects meta-analysis. Because loss to follow up in HIV programmes is known to include some deaths, we used attrition as our primary outcome, defined as death plus loss to follow-up. We assessed evidence quality with GRADE methodology.

**MAIN RESULTS:**

Sixteen studies met the inclusion criteria, all but one were from Africa, comprising two cluster randomised trials and 14 cohort studies. Antiretroviral therapy started at a hospital and maintained at a health centre (partial decentralisation) probably reduces attrition (RR 0.46, 95% CI 0.29 to 0.71, 4 studies, 39 090 patients, moderate quality evidence). There may be fewer patients lost to care with this model (RR 0.55, 95% CI 0.45 to 0.69, low quality evidence). We are uncertain whether there is a difference in attrition for antiretroviral therapy started and maintained at a health centre (full decentralisation) compared to a hospital at 12 months (RR 0.70, 95% CI 0.47 to 1.02; four studies, 56 360 patients, very low quality evidence), but there are probably fewer patients lost to care with this model (RR 0.3, 95% CI 0.17 to 0.54, moderate quality evidence). When antiretroviral maintenance therapy is delivered at home by trained volunteers, there is probably no difference in attrition at 12 months (RR 0.95, 95% CI 0.62 to 1.46, two trials, 1453 patients, moderate quality evidence).

**AUTHORS' CONCLUSIONS:**

Decentralisation of HIV care aims to improve patient access and retention in care. Most data were from good quality cohort studies but confounding between site of treatment and outcomes cannot be excluded. Nevertheless, this review found that attrition appears to be lower in partial decentralisation models of treatment, where antiretrovirals were started at hospital and continued in the health centre; with antiretroviral drugs started and continued at health centres, no difference in attrition was detected, but there were fewer patients lost to care. For antiretroviral therapy provided at home by trained volunteers, no difference in outcomes were detected when compared to facility-based care.

**This is the abstract of a Cochrane Review published in the Cochrane Database of Systematic Reviews (CDSR) 2013, issue 6. Art. No.:** CD009987. DOI: 10.1002/14651858.CD009987.pub2 (http://onlinelibrary.wiley.com/doi/10.1002/14651858.CD009987.pub2/abstract). For full citation and authors' details, see reference 1.

**The full text of this review is freely available from:** http://onlinelibrary.wiley.com/doi/10.1002/14651858.CD009987.pub2/pdf. 
